# Enhancement of β-xylosidase productivity in cellulase producing fungus *Acremonium cellulolyticus*

**DOI:** 10.1186/2191-0855-1-15

**Published:** 2011-06-30

**Authors:** Machi Kanna, Shinichi Yano, Hiroyuki Inoue, Tatsuya Fujii, Shigeki Sawayama

**Affiliations:** 1 Biomass Technology Research Center, National Institute of Advanced Industrial Science and Technology, 3-11-32 Kagamiyama, Higashi-Hiroshima, Hiroshima, 739-0046 Japan; 2 Graduate School of Agriculture, Kyoto University, Kitashirakawa Oiwake-cho, Sakyo-ku, Kyoto, 606-8502, Japan

**Keywords:** Hemicellulase, beta-xylosidase, *Acremonium cellulolyticus*, Transformation, Cellulase

## Abstract

Enzymatic hydrolysis is one of the most important processes in bioethanol production from lignocellulosic biomass. *Acremonium cellulolyticus *is a filamentous fungus with high cellulase production but productivity of hemicellulase, especially β-xylosidase, is lower than other filamentous fungi. We identified 2.4 Kb β-xylosidase gene in the *A. cellulolyticus *genome sequence information and it encoded 798 amino acids without introns. To enhance hemicellulase productivity in *A. cellulolyticus*, we transformed this fungus with the identified β-xylosidase gene driven by the *cellobiohydrolase Ι *(*cbh1*) promoter, using the protoplast-polyethyleneglycol (PEG) method, and obtained a transformant, YKX1. Hydrolysis rate of xylooligosaccharides was more than 50-fold higher using culture supernatant from YKX1 than that from the parental strain, Y-94. Total cellulase activity (measured by filter paper assay) in YKX1 was not affected by the *cbh1 *promoter used for expression of β-xylosidase, and induced by cellulose. Since YKX1 can produce larger amount of β-xylosidase without affecting cellulase productivity, it is considered to be beneficial for practical monosaccharide recoveries from lignocellulosic biomass.

## Introduction

Ethanol produced from lignocellulosic biomass is a second-generation biofuel which does not compete with food resources (Sims et al. 2010) and is expected to be an alternative to gasoline that reduces dependence on fossil fuels. Bioethanol can be produced from lignocellulosic biomass *via *several processes; pretreatment, enzymatic hydrolysis, and fermentation. In these processes, effective enzymatic hydrolysis of cellulose and hemicellulose is the most important step. Therefore, we focused on the enzyme activities that catalyze the saccharification of cellulose and hemicellulose.

Cellulose is the primary component of lignocellulosic biomass. After cellulose, hemicellulose is the second most abundant component of the plant cell wall, and accounts for 20-30% of lignocellulosic biomass (Girio et al. 2010). Glucuronoxylans (O-acetyl-4-O-methylglucuronoxylan) are the most abundant type of hemicellulose, and they make up 15-30% of the dry mass in hardwoods (Girio et al. 2010). Although conventional ethanol fermenting yeasts cannot utilize xylose, we have developed efficient xylose-fermentable *Saccharomyces cerevisiae *strains (Matsushika et al., 2009). Hence, effective saccharification of xylan is practically important for attaining higher yields of monosaccharides.

Endo-β-1, 4-xylanase and β-xylosidase catalyze the production of xylooligosaccharides from xylan and xylose from xylooligosaccharides, respectively. β-xylosidase hydrolyzes the non-reducing end of xylooligosaccharides. Although purified β-xylosidase is commercially available, it is too costly for practical large-scale applications. It will be beneficial if cellulase-producing filamentous fungi also produce hemicellulase for efficient and cost-effective hydrolysis of lignocellulosic biomass.

The β-xylosidase genes have been sequenced and characterized from many species of filamentous fungi. *xylA *of *Aspergillus oryzae *(Kitamoto et al. 1999) and *xyl I *of *Aureobasidium pullulans *strain ATCC 20524 (Ohta et al. 2010) belong to the glycosyl hydrolase (GH) 3 family. *xylB *in *A. oryzae *belongs to the GH43 family (Suzuki et al. 2010). Based on amino acid sequence, *Bxl1 *of *Trichoderma reesei *RutC-30 (Margolles-Clark et al. 1996), *XlnD *of *Aspergillus nidulans *(Perez-Gonzalez et al. 1998), and *Aspergillus niger *(van Peij et al. 1997) belong to the GH 3 family (Suzuki et al. 2010). Furthermore, heterologous expression of *xylB *of *A. oryzae *in *Escherichia coli *was more stable than endogenous *XylB *expression in *A. oryzae *(Suzuki et al. 2010) or heterologous expression of *xlnD *from *A. **n**iger *strain ATCC 10864 in *Aspergillus awamori*, which has similar activity on 4-nitrophenyl-β-D-xyloopyranoside to *A. niger XlnD *(Selig et al. 2008).

*Acremonium cellulolyticus *is a cellulase-producing filamentours fungus isolated in Japan (Yamanobe et al., 1987). Repeated UV and/or nitrosoguanidine (NTG) mutagenesis of the wild strain Y-94 was used to enhance cellulase productivity, and strains with high cellulase productivity (TN, C-1, and CF-2612) have been selected. Although the productivity of cellulase is quite high in *A. cellulolyticus*, its hemicellulase production is not sufficient. Recently, we have sequenced the whole genome of *A. cellulolyticus *(unpublished data) and could deduce many genes for saccharifying enzymes. Although there are only a few reports of successful transformation in *A. cellulolyticus*, we could successfully obtain many transformants with protoplast- PEG method. Therefore, we tried to enhance β-xylosidase productivity of this fungus by introducing its β-xylosidase gene under strong promoter.

Among strains of *A. cellulolyticus*, CF-2612 has the highest cellulase productivity (Fang et al. 2009), but it underwent random mutagenesis and may have mutations at every site as well as cellulase related genes. And apparently random mutagenesis affected the growth rates of CF-2612 because it grows more slowly than other strains. Therefore, we used the wild type strain, Y-94, in our study.

## Materials and methods

### Fungal strain and culture condition

*A. cellulolyticus *Y-94 (FERM Number BP-5826) was cultured in 10 ml of medium in 100 ml flasks at 30°C with shaking at 200 rpm. The composition of the culture medium for *A. cellulolyticus *was described previously (Fang et al. 2009). Sampling was performed at 1, 3, and 7 days for analysis of gene expression using real-time PCR and/or enzyme activity. *A. cellulolyticus *strains were cultured in potato dextrose (PD) medium for cloning and transformation.

### Measurement of the amount of ATP, β-xylosidase and β-mannosidase activity, and saccharification efficiency

The amount of ATP measured based on fluorescence using a Rucifel-250 kit (Kikkoman, Tokyo Japan) and Lumitester C-100 (Kikkoman) according to the manufacturers' instructions.

Activities for β-xylosidase and β-mannosidase were measured using 100 μl of 10 mM 4-nitrophenyl-β-D-xyloopyranoside (PNP-Xyl, Sigma, MO USA) or 4-nitrophenyl-β-D-mannopyranoside (PNP-Man, Sigma) as the substrates (final concentration is 1 mM), respectively. Fifty μl of enzymes were incubated with 1 mM PNP-Xyl or PNP-Man at 45°C for 10 minutes in 850 μl of 50 mM acetic acid buffer (pH.5). After 10 minutes, 500 μl of 1 M NaCO_3 _was added. Because 4-nitrophenol which will be generated from substrates by enzymatic hydrolysis is a chromogenic substance, enzyme activities were assayed by measuring absorbance at 420 nm by UV-2550 Spectrophotometer (Shimadzu, Kyoto, Japan). One unit of the enzyme activity is defined as the amount of enzyme that produces 1 μmol of p-nitrophenol per minute. For analysis of saccharification efficiency, culture medium was centrifuged at 9,000 g for 10 min to collect the supernatant containing the secreted enzyme. Enzyme solutions were incubated at 45°C in 50 mM acetic acid buffer with 4% xylooligosaccharides (Wako Pure Chemicals, Osaka JAPAN). The xylose concentration was measured using a high performance liquid chromatography system (JASCO, Tokyo, Japan), under the conditions described previously (Buaban et al. 2010).

### Cloning β-xylosidase gene from *A. cellulolyticus*

A putative β-xylosidase gene, *bxy3A*, was identified in *A. cellulolyticus *genome sequence information using *A. nidulans xlnD *sequence as the query for a homology search. In silico molecular cloning (in silico biology, Yokohama Japan) which is a software for gene analysis was used for homology search. Augstus 2.2 http://augustus.gobics.de/ which is a program for eukaryotic genome sequence was used for the prediction of genes. The β-xylosidase coding region was amplified using *A. cellulolyticus *CF-2612 genomic DNA as the template, and the *cellobiohydrolase Ι *(*cbh1*) promoter was amplified from Y-94 genome. For the extraction of genomic DNA, cells cultured in PD medium were collected by centrifugation, and 3 volumes of TE (10 mM Tris-HCl, 1 mM EDTA, pH 8.0) with 2% sodium dodecyl sulfate (SDS) were added to the cell pellet. The cell suspension was incubated at 50°C for 1 hr. Potassium acetate (5 M) was added to the cell suspension at one-tenth of total volume, and this mixture was incubated on ice for 1 hr. The mixture was centrifuged at 13,000 g for 10 min, and the supernatant was subjected to two rounds of phenol-chloroform treatment, and ethanol precipitation was performed to obtain genomic DNA. The DNA was incubated with RNaseA (Nippon gene, Toyama, Japan) at 37°C for 1 hr to degrade contaminating RNA.

To amplify β-xylosidase open reading frame from *A. cellulolyticus *DNA, the forward primer with engineered *Spe*Ι site (5'-GCACTAGTATGGTCTACACCACG) and the reverse primer with engineered *Kpn*Ι site (5'-GCGGTACCTCAATTAGAATCAGGC) were designed based on sequence from the *A. cellulolyticus *genome sequence information (unpublished data) using Augustus 2.2 software The promoter from the cellobiohydrolase Ι (*cbh1*) gene (GenBank Accession number; E39854) was amplified with the forward primer with an *Xho*Ι site (5'-GCCTCGAGAAGCTTGGAAGCT) and the reverse primer with a *Spe*Ι site (5'-TACCATGGCTGCACTAGTGTGTCGATTGCTT). The amplified fragment of the *cbh1 *promoter was connected to β-xylosidase gene in frame, and incorporated into a shuttle vector pLD10 provided by Dr. H. Corby Kistler (University of Minnesota, USA), and the resulting plasmid, pLcbX-1, was obtained. *E. coli *DH5α cells (Takara Bio, Shiga, Japan) were used to maintain the plasmid.

### Transformation of *A. cellulolyticus*

The parental strain, Y-94, was transformed using a slightly modified protoplast-PEG method (Fincham et al. 1989). An overnight culture of *A. cellulolyticus *was treated with in 10 mM KH_2_PO_4_, 0.8 M NaCl, and 0.2% Yatalase (Takara Bio) to prepare protoplasts. The protoplasts were washed with 0.8 M NaCl and suspended in Solution A (1.2 M Sorbitol, 10 mM Tris-HCl, 10 mM CaCl_2_). Plasmid (10 μg) was added to the protoplast suspension, then 50 μl of Solution B (40% PEG4000, 10 mM Tris-HCl, 10 mM CaCl_2_) was added to the protoplast suspension, and the suspension was incubated on ice for 30 min and RT for 15 min. Then, 8.5 ml of solution A was added to the cell suspension to dilute Solution B. The protoplasts suspension was spread on YPSA plate (1% Bacto yeast extract, 1% Bacto tryptone, 1 M Sucrose, and 2% Agar) and incubated overnight at 30°C. PD medium with 0.2% agar with 500 μg hygromycin was piled on to the YPSA plate. A single colony was isolated 3 days after the addition of PD medium. To confirm the presence of *hygromycin phosphotransferase *(*hph*) gene, transformant was checked by PCR using the forward (5'-ATGCCTGAACTCACCGCGAC-3') and the reverse (5'-CTATTCCTTTGCCCTCGGAC-3') primers.

### Measurement of FPU, xylanase, and mannanase activities

The FPU activity assay described by Ghose (1987) was performed as a reference for cellulase activity. Whatman NO.1 filter paper (paper size; 1 cm × 6 cm, Whatman, Kent UK) was used as the substrate. Culture medium was centrifuged at 9,000 g for 10 min to collect the supernatant with the secreted enzyme. Enzyme solution in 1 ml of 50 mM citric acid buffer, pH4.8, was incubated with the substrate at 50°C for 60 min. DNS solution (3 ml; 1% 3, 5-dinitrosalycilic acid (Sigma), 1.2% NaOH, 0.05% sodium sulfate, and 20% potassium sodium tartrate tetrahydrorate) was added to the enzyme solution, and the mixture was boiled for 5 min, then the reaction mixture was put on ice. The amount of glucose was measured with the absorbance at 540 nm with UV-2550 Spectrophotometer as a reducing sugar.

For assay of xylanase and mannanase, 2% birch-wood xylan (Sigma) or 1% Konjac Glucomannan (Megazyme, Wicklow, Ireland) were used as substrates, respectively. The incubation time for enzymatic reaction was 30 min and activities were assayed according to the previously described method (Bailey et al. 1991).

### Measurement of gene expression by real-time PCR

For analyzing expression of the β-xylosidase gene, we performed real-time PCR. RNA was extracted with Fast RNA Pro Red kit (MP biomedicals CA USA) at one day after starting the cultures. The extracted RNA was cleaned using the RNeasy mini kit (Qiagen, Hilden Germany). cDNA was prepared using the M-MLV reverse transcriptase (Takara Bio) and oligo (dT) 20 (Toyobo, Osaka Japan). Samples were labeled by iQ SYBR Green (Bio-Rad, CA USA). Primers used for real-time PCR were as follows: For β-xylosidase: 5'-TTCCCGGTTAGGGTTTGATG-3' (forward) and 5'-GGGACACCATTCACCGAGTT-3' (reverse), for cellobiohydrolase I: 5'-ACTGCCTCCTTCAGCAAACAC-3' (forward) and 5'-GGCGTAGTCGTCCCACAAA-3' (reverse), for β-actin (the internal control): 5'-CAACTGGGACGACATGGAGA-3' (forward) and 5'-GTTGGACTTGGGGTTGATGG-3' (reverse).

### Nucleotide sequence accession number

The DDBJ accession number of *bxy3A *sequence is AB613265.

## Results

A putative β-xylosidase gene, *bxy3A*, was identified in *A. cellulolyticus *genome sequence information using the *A. nidulans xlnD *sequence as the query for a homology search. The size of the gene was 2.4-Kb and it encoded 798 amino acids without introns. The amino acid sequence deduced from *bxy3A *was aligned with other β-xylosidase sequences (data not shown). This protein had 74% identity to *T. reesei *Bxl1 (Margolles-Clark et al. 1996) and 61% identity to *A. nidulans *XlnD (Perez-Gonzalez et al. 1998).

**Figure 1:**
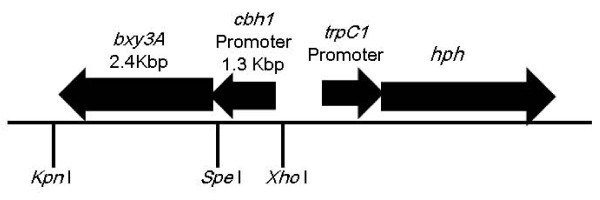
**The structure of the expression cassette in transformation vector pLcbx-1**. The deduced β-xylosidase gene was ligated at the indicated restriction site. *TrpC*; Transient receptor potential *hph*; hygromycin phosphotransferase

The Bxy3A expression construct is shown in Figure 1. The *cbh1 *promoter was used to drive expression of the deduced *bxy3A *open reading frame. The *hph *gene coding region driven by the transient receptor potential (trp) C promoter was used as the selection marker. The plasmid, pLcbX-1, with the *Bxy3A *expression construct and the *hph *marker was transformed into *A. cellulolyticus*Y-94 using the protoplast-PEG method.

**Figure 2:**
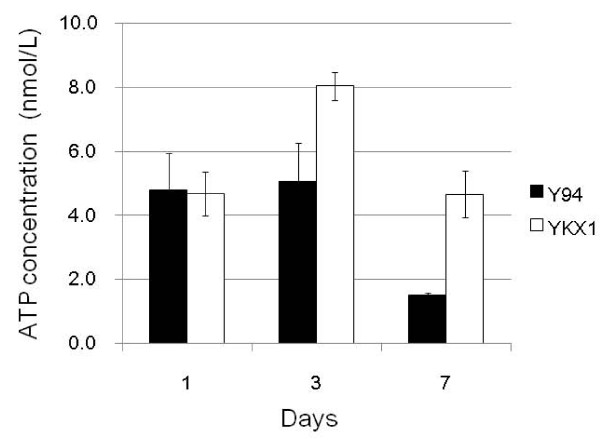
**The amount of ATP as the indicator of fungal growth at 1, 3, and 7 days**. Closed column; Y-94, Open column; YKX1. Standard deviations are calculated from triplicate experiments.

We had transformation experiments in *A. cellulolyticus *with protoplast-PEG method using *hph *as the selection marker, and could obtain transformants. The mean value of transformation efficiency was 0.24 × 10^6 ^cells^-1^. We have isolated a transformed colony with pLcbX-1on a YPSA plate containing 500 μg/ml hygromycin. The transformant, YKX1, could grow on PDA plates with 500 μg/ml hygromycin, and the presence of *hph *was confirmed by PCR from genomic DNA of the strain (data not shown). Growth rates were determined by measuring ATP concentration, because growth rates of filamentous fungi cannot be well assessed by measuring optical density. The growth of YKX1 gradually increased relative to that of Y-94 by 3 and 7 days after the start of the cultures, though growth rates were similar on the first day after the start of the cultures (Figure 2).

**Figure 3:**
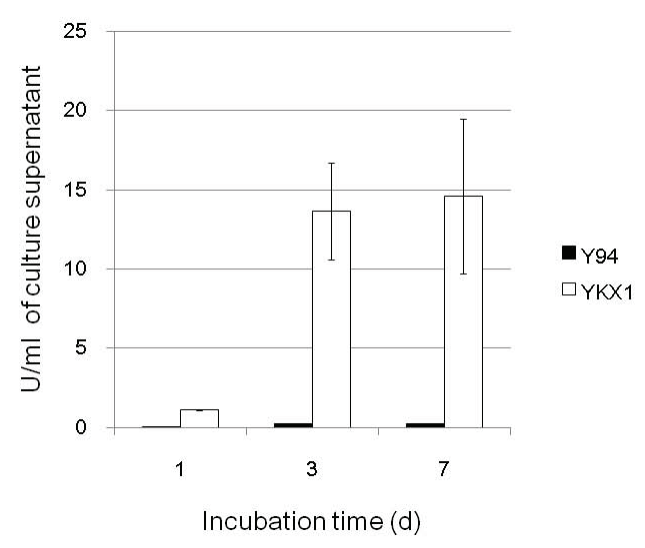
**Activity of β-xylosidase was measured 1, 3, and 7 days after culture grown in 4% solca flock**. Closed column; Y-94, Open column; YKX1. Standard deviations are calculated from triplicate experiments.

We measured β-xylosidase activity in YKX1 and Y-94 cultured in medium with cellulose as a sole carbon source, on days 1, 3 and 7. The β-xylosidase activity was markedly higher in YKX1 than in Y-94 (Figure 3). The amount of secreted protein in YKX1 was similar to that in Y-94 on all days (data not shown). To assess *bxy3A *gene expression in YKX1 and Y-94, we performed real-time PCR (Table 1). *bxy3A *expression was more than ten-fold higher in YKX1 than in Y-94 when the strains were grown in medium with cellulose. Although the expression of *cbh1 *was slightly lower on average in YKX1 than in Y-94, FPU values of Y-94 (1.27 ± 0.14 FPU/ml) and YKX (1.13 ± 0.15 FPU/ml) were not significantly different. This fact suggests that the *cbh1 *gene was not disrupted by homologous recombination. We analyzed the insertion position by the genome walking method (data not shown). The *cbh1 *was located at HND05_CDS0018, but the transformation cassette was not inserted at either of this position (data not shown).

**Table 1:** Gene expression in Y94 and YKX1 was analyzed one day after the cultures were started.

	Y94	YKX1
β-xylosidase	0.02 ± 0.004	3.3 ± 0.509
Cellobiohydrolase I	15.78 ± 3.316 *	11.16 ± 2.321 *

Number showed expression relative to an internal control (gene/β-actin)

*P < 0.01

**Figure 4:**
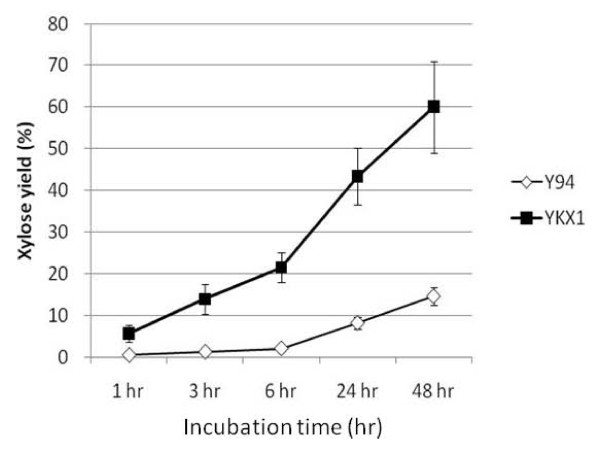
**Xylose yields from saccharification of xylooligosaccharides**. The enzyme solution was prepared from culture medium with 4% solca flock. Closed symbol; Y-94, Open symbol; YKX1 Standard deviations are calculated from triplicate experiments.

We also measured xylanase activity both in YKX1 and Y-94, and activity was similar in YKX1 and Y-94 on the third day of culture (Table 2). Furthermore, activities of other hydrolyzing enzymes i.e. β-mannanse and β-mannosidase were also similar in YKX1 and Y-94 (Table 2). We had experiments of xylooligosaccharides hydrolysis of the transformant. After 1 hr incubation, YKX1 cultures had a higher xylose yield than did Y-94 cultures (Figure 4). After 48 hr, YKX1 cultures had xylose yield of 60% from xylooligosaccharides.

**Table 2:** Enzyme activity 3 days after the cultures were started.

	Y94 (U/ml)	YKX1 (U/ml)
β-xylanase	56.68 ± 4.13	59.59 ± 2.96
β-mannanase	4.21 ± 0.29	3.76 ± 0.21
β-mannnosidase	0.0071 ± 0.00042	0.0063 ± 0.00042

## Discussion

In filamentous fungi, β-xylosidase of each species belongs to one of 3 GH families, 3, 43, or 54 (Knob et al. 2010). The putative β-xylosidase gene in *A. cellulolyticus*, *bxy3A*, showed high homology to β-xylosidase of the GH 3 family, and Bxy3A shared a conserved motif with the GH 3 family.

In this study, to increase hemicellulase productivity, the *cbh1 *promoter was used to drive overexpression of BXY3A. CBH1, which is one of major cellulase, cleaves cellulose at the non-reducing end and produces cellobiose. CBH1 protein accounts for about 60% of all secreted proteins in *T. reesei*. Therefore, the *cbh1 *promoter is widely used as a promoter for overexpression of homologous or heterologous gene (Keränen and Penttilä 1995). However, expression cassettes containing the *cbh1 *promoter might integrate at the *cbh1 *loci by homologous recombination. Actually, it was reported that expression of *cbh1 *was abolished by homologous recombination, pcbh1-gus in *T. reesei *PC-3-7 (Rahman et al. 2009). Moreover, the value of cellulase activity of Pcbh1-gus, in which *uidA *encoding β-glucuronidase is ligated between the *cbh1 *promoter and the *cbh1 *terminator, was half of that in wild type (Rahman et al. 2009). In this study, FPU activity of YKX1 was similar to that of Y-94. In most transformation experiments in filamentous fungi, a terminator is added downstream of the open reading frame. We did not ligated the terminator of *cbh1 *at downstream of *bxy3A*. Therefore, the expression cassette was less likely to induce a double crossover. However, it is possible that a single crossover could have occurred at *cbh1 *gene. Although *cbh1 *gene of YKX1 was slightly lower than that of Y-94, expression cassette did not disrupt the original *cbh1 *gene.

β-xylosidase gene in the integrated transformation cassette was expressed and the produced enzyme functioned. Xylosidase activity in YKX1 was higher than that in Y-94 grown in PD medium (data not shown), while activity of YKX1 was much higher than that of Y-94 in medium with 4%Solcaflock (Figure 3). Another potential problem with using the *cbh1 *promoter was a possible titration effect; the cellulase genes may have bound most of the transcription factor, *xyr1*, in *T. reesei *(Stricker et al. 2006). In *A. niger*, *xlnR *which is an ortholog of *xyr1*, induces expression of the cellulase gene (Stricker et al. 2008). In both cases, expression from the endogenous *cbh1 *gene might decrease by an additional *cbh1 *promoter due to competition for the transcription factor. However, we found that FPU activity of YKX1 was similar to Y-94, which suggests titration effect did not occur in YKX1.

We were also concerned that a titration effect might decrease expression from the endogenous β-xylosidase gene because *xyr1 *induces *bxl1 *and *xlnR *induces *xlnD *in *T. reesei *and *A. niger*, respectively (Stricker et al. 2006, Stricker et al. 2008). It was difficult to determine whether the endogenous β-xylosidase was influenced by a titration effect because YKX1 was transformed with the open reading frame from the endogenous β-xylosidase gene. However, expression of *bxy3A *driven by the *cbh1 *promoter in YKX1 was higher than expression of the endogenous *bxy3A *in the parental strain (Table 1). Because there was no evidence of any titration effect in YKX1, it is appropriate to use the *cbh1 *promoter to enhance hemicellulase activity in the strain *A. cellulolyticus *Y-94.

Expression of the xylanolytic gene, *bxl1*, is induced by xylobiose in *T. reesei *(Margolles-Clark et al. 1997). In *A. nidluns*, D-xylose induces xylanolytic enzymes *via *the regulatory gene, *xlnR *(Tamayo et al. 2008). Although D-xylose and xylobiose can induce xylanolytic enzymes, another report indicates that the activity of β-xylosidase depends on the concentration of D-xylose in *T. reesei *(Mach-Aigner et al. 2010). β-xylosidase activity of YKX1 might have no effect in the presence of D-xylose because YKX1 used cbh1 promoter.

YKX1 grew faster than Y-94 in the same medium measured by ATP amount (Figure 2). In a previous study, cellulase activity increased gradually as the amount of ATP decreased (Fang et al. 2009). Similarly in YKX1, xylosidase activity was highest at 7 days, which was when ATP concentrations decreased. The behavior of the enzymatic activity was similar to other cellulolytic enzyme in this previous report (Fang et al. 2009) because the promoter used was from cellulolytic enzyme expression. Furthermore, the amount of ATP in YKX1 was higher than that in Y-94 at 3 and 7 days (Figure 2). In contrast, the values of cellulase activity in CF-2612 and C-1 are negatively correlated ATP concentrations (Fang et al. 2009). However, ATP levels in YKX1 were consistently high than those in Y-94.

Furthermore, the activities of other hemicellulolytic enzymes were similar in YKX1 and Y-94 (Table 2), indicating that the transformation was not affected by other hemicellulase. We generated a transformant of *A. cellulolyticus *that has higher β-xylosidase productivity than the parental strain without affecting cellulase or other hemicellulase productivity. We confirmed xylose yield improved by adding enzyme solution of YKX1, in saccharification experiments using rice straw (data not shown). Therefore, YKX1 should be useful for enzyme production in practical applications that convert both cellulose and xylan into fementable monosaccharides.

## Competing interests

The authors declare that they have no competing interests.
